# Personalized Medicine for Neuroblastoma: Moving from Static Genotypes to Dynamic Simulations of Drug Response

**DOI:** 10.3390/jpm11050395

**Published:** 2021-05-11

**Authors:** Jeremy Z. R. Han, Jordan F. Hastings, Monica Phimmachanh, Dirk Fey, Walter Kolch, David R. Croucher

**Affiliations:** 1 Garvan Institute of Medical Research, Sydney, NSW 2010, Australia; j.han@garvan.org.au (J.Z.R.H.); j.hastings@garvan.org.au (J.F.H.); m.phimmachanh@garvan.org.au (M.P.); 2 Systems Biology Ireland, School of Medicine, University College Dublin, Belfield, Dublin 4, Ireland; dirk.fey@ucd.ie (D.F.); walter.kolch@ucd.ie (W.K.); 3 Conway Institute, University College Dublin, Belfield, Dublin 4, Ireland; 4 St Vincent’s Hospital Clinical School, UNSW Sydney, Sydney, NSW 2052, Australia

**Keywords:** neuroblastoma, personalized medicine, chemotherapy, dynamic modelling, patient-specific modelling, apoptosis

## Abstract

High-risk neuroblastoma is an aggressive childhood cancer that is characterized by high rates of chemoresistance and frequent metastatic relapse. A number of studies have characterized the genetic and epigenetic landscape of neuroblastoma, but due to a generally low mutational burden and paucity of actionable mutations, there are few options for applying a comprehensive personalized medicine approach through the use of targeted therapies. Therefore, the use of multi-agent chemotherapy remains the current standard of care for neuroblastoma, which also conceptually limits the opportunities for developing an effective and widely applicable personalized medicine approach for this disease. However, in this review we outline potential approaches for tailoring the use of chemotherapy agents to the specific molecular characteristics of individual tumours by performing patient-specific simulations of drug-induced apoptotic signalling. By incorporating multiple layers of information about tumour-specific aberrations, including expression as well as mutation data, these models have the potential to rationalize the selection of chemotherapeutics contained within multi-agent treatment regimens and ensure the optimum response is achieved for each individual patient.

## 1. Introduction

Neuroblastoma is a paediatric malignancy of the sympathetic nervous system, accounting for 7–8% of childhood cancers [1]. While it is relatively rare, it is the most commonly diagnosed extra-cranial cancer during infancy and disproportionally accounts for 12–15% of cancer-related deaths in this age group [1,2]. Neuroblastoma manifests anywhere along the peripheral sympathetic nervous system, predominantly in the adrenal medulla and the sympathetic ganglion [3]. Histologically, it presents as a highly heterogeneous tumour of small round blue cells, with varying degrees of differentiation. This inherent heterogeneity is also reflected in the diverse clinical behaviour of these tumours, spanning from spontaneous tumour regression to progression of metastatic disease [1].

Promisingly, patient outcomes for neuroblastoma have improved substantially since the early 1970s, with 5-year disease-free survival increasing from 50% to 75% in 2005 [4], and to 81% as of 2020 [5]. While some of the improvement in patient outcome is attributed to early diagnosis, refined risk stratification and the development of new treatment regimens, it is mostly due to increased cure rates for low- and intermediate-risk neuroblastoma. In these patients, tumours are often only observed and where possible surgically resected, with a subsequent 5-year survival greater than 98% and 90–95%, respectively [1]. Very rarely, these patients will also require low doses of single-agent chemotherapy [6] (Table 1). However, 70% of patients are diagnosed with high-risk disease and frequently present with unresectable metastatic disease. The 5-year survival rate for high-risk neuroblastoma is less than 50%, despite multi-agent and multi-modal therapy [7] (Table 1). Approximately 15% of all high-risk patients will not respond to initial therapy [8] and overall 40–50% of high-risk patients will relapse [4,8,9]. In these recurrent tumours, overall survival is less than 10% [3].

Most neuroblastomas present with no obvious genetic predisposition or family history, and a generally low somatic mutation profile [10,11]. Therefore, little progress has been made in the development of targeted therapies, with recent therapeutic advances limited to disialoganglioside (GD2) immunotherapy, small molecule anaplastic lymphoma kinase (ALK) inhibitors and metaiodobenzylguanidine (MIBG) radiotherapy [12,13]. As such, chemotherapy remains the backbone for neuroblastoma treatment. Tragically, neuroblastoma patients who are cured are often at risk of secondary complications arising from exposure to genotoxic chemotherapy at such a young age, including infertility, bone necrosis, deafness and the development of secondary cancers [14]. Evidently, there is an urgent need to not only develop new treatment options, but to also develop more finely tailored approaches for the treatment of high-risk neuroblastoma patients with optimized regimens that can incorporate both standard-of-care chemotherapy and emerging drugs.

Therefore, this review will provide a brief overview of the molecular and genetic landscape of neuroblastoma, along the relevant targeted therapies in current use and their potential limitations. Building on this, we will further explore the potential for patient-specific simulations of drug response to be developed into a personalized medicine approach applied to the treatment of all high-risk neuroblastoma patients with rationalized combinations of standard-of-care chemotherapy drugs.

**Table 1:** Current standard-of-care therapy for neuroblastoma based on the Children’s Oncology Group neuroblastoma risk stratification groups.

Risk	Disease Description	Treatment	Drug Regimen	Survival (5y)	Ref. [6]
Low	Localized tumour	Most tumours will regress. Debulking surgery is sometimes required, and most patents do not receive chemotherapy	N/A	>98%	[15]
Intermediate	Localized tumour	Debulking surgery and moderate-intensity chemotherapy	4–8 cycles of: Cisplatin, etoposide, cyclophosphamide or doxorubicin	90–95%	[15]
High	Metastatic disease of bone and bone marrow	High-intensity induction therapy with the aim of shrinking tumours with: ∙ Autologous stem cell harvest and transplantation Surgical resection ∙ Radiation therapy ∙ Immunotherapy (GD2) ∙ Cis-retinoic acid	Induction with 2 cycles of high doses of either: ∙ Topetecan and cyclophosphamide ∙ Or vincristine, cyclophosphamide and doxorubicin	40–50%	[16]
		Salvage therapy for refractory or relapsed tumours	Combinations of: ∙ Irinotecan with temozolomide ∙ topetecan with cyclophosphamide	<10%	[17,18]
Special (4S)	Prone to spontaneous regression (with potential metastatic liver and skin lesions)	Debulking surgery with a mainly “wait and see” approach Disease with liver metastasis: ∙ Low-intensity chemotherapy ∙ External bean radiation	Cisplatin, etoposide, cyclophosphamide, doxorubicin	>90%	[15]

## 2. Molecular Landscape and Opportunities for Targeted Therapy

Seminal genomic studies have shed light on the underlying tumour landscape of neuroblastoma, although like many paediatric malignancies, neuroblastomas usually harbour fewer somatic genomic aberrations and mutations than adult tumours [19,20]. Furthermore, genome wide studies have since confirmed that no single genetic aberration is responsible for the development of all neuroblastomas [19,20,21]. Instead, sporadic disease is driven by the combination of multiple low frequency mutations and deleterious chromosomal events. To date, *ALK* mutations and *MYCN* amplification are the only validated de novo drivers of neuroblastoma [22,23], although a number of other commonly over-expressed proteins and low frequency somatic mutations have been implicated in tumour progression and drug resistance. The potential for these tumour specific aberrations to be therapeutically targeted within a personalized medicine paradigm is further discussed below.

### 2.1. MYCN 

The MYC family of transcription factors regulate multiple cellular processes including apoptosis, proliferation, the cell cycle, mitochondrial function and glycolysis [24]. *MYCN* amplification occurs in ~20% of neuroblastomas and has been established as a key driver of an aggressive and chemoresistant tumour phenotype, frequently observed in high-risk neuroblastoma and predictive of poor patient outcome [20,25]. Early in vitro models demonstrated elevated MYCN expression induced neuroblastoma tumour growth and proliferation [26], while transgenic mouse [22,27] and zebrafish [28] models with induced MYCN expression underwent spontaneous neuroblastoma formation.

Frustratingly, while overexpression of MYCN is a known major driver of disease, it also represents a potentially difficult avenue for therapeutic targeting in the context of a personalized medicine approach. The lack of targetable surfaces in its DNA binding domain and conserved homology among the MYC family proteins has meant that efforts aimed at direct MYCN inhibition have thus far been ineffective [29]. Alternative approaches, including those involving the inhibition of MYCN hetero-dimerization with MAX homodimers have been proposed as inhibitory strategies capable of reducing MYCN transcriptional activity [30,31]. The best known example is the compound 10058-F4, a c-Myc inhibitor that also prevented MYCN/MAX hetero-dimerization in vitro [32], inhibited tumour growth and improved survival in a MYCN transgenic mouse model of neuroblastoma [33]. These results suggest that targeting MAX may warrant further clinical investigation. More promising approaches have targeted the transcriptional machinery necessary for MYCN to exert its oncogenic functions. This has included the BET family of proteins, which are involved in the transcriptional regulation of multiple genes such as *MYCN*—with BET inhibitors JQ1 and OTX015 being considered for clinical evaluation [34]. Additionally, binding partners of MYCN such as Aurora A kinase (AURKA), have been targeted with alisertib in combination with current standard-of-care chemotherapy in multiple phase 2 trials [35,36], while many other indirect methods of inhibition of MYCN are currently being evaluated and are more thoroughly covered in other reviews [13,37].

### 2.2. ALK

ALK is a member of the insulin receptor tyrosine kinase (RTK) superfamily, for which high expression levels are usually confined to neurons during nervous system development, although it can also be highly expressed in neuroblastoma tumours [38]. *ALK* mutations are the most frequently observed somatic mutations in neuroblastoma, with either point mutations, amplification or fusion events occurring in 7–10% of cases [39,40].

Given mutant ALK has been successfully targeted in other diseases such as in anaplastic large-cell lymphoma, non-small-cell lung cancer and myofibroblastic sarcoma, ALK has been heavily investigated as a therapeutic target in neuroblastoma [41]. ALK inhibitors bind to the ATP binding pocket of ALK, preventing auto-phosphorylation and downstream signal transduction [37]. To date, ALK inhibitors such as crizotinib and entrecenib have shown efficacy in preclinical models and progressed to phase 1 and 2 trials for relapsed and refractory neuroblastoma [42,43]. Off-target effects and acquired resistance has limited the clinical applications of these inhibitors, resulting in the development of second- and third-generation inhibitors including ceritinib [44] and lolatinib [45], which are recruiting or starting phase 1 trials. Many of these clinical trials have shown that ALK inhibition also sensitized tumours to standard-of-care chemotherapy, supporting the combination of ALK inhibitors with current standard-of-care treatments for the small number of patients with ALK mutant neuroblastoma.

### 2.3. Trk Receptor Family

The tropomyosin receptor kinase family, TrkA, TrkB, TrkC (encoded by *NTRK1*, *NTRK2*, *NTRK3*), are RTKs with established spatiotemporal roles in the development and maintenance of the peripheral nervous system [46]. TrkA is expressed during the differentiation of progenitor cells into sympathetic neurons, while less differentiated neurons tend to express TrkC and infrequently, TrkB [47,48]. Differential expression of these RTK’s has been implicated in neuroblastoma, although with opposing functional and prognostic outcomes [49]. Tumours with high TrkA and TrkC expression are prone to differentiation and spontaneous regression, and thus predictive of a favourable clinical outcome [50,51,52,53]. Conversely, high mRNA expression of TrkB and its ligand BDNF is observed in 36% and 68% of high-risk disease cases, respectively [54], and is significantly associated with poor prognosis [51,55,56]. Trk receptors are therefore considered an attractive target for targeted therapies, with the selective Trk inhibitor, lestaurtinib, and pan-ALK/Trk/ROS1 inhibitor, entrectinib, showing favourable preclinical and phase 1 clinical results [57,58] for relapsed/refractory neuroblastoma tumours.

### 2.4. Other Genetic Aberrations

Interestingly, many patients without MYCN amplification also have poor prognosis, implicating the role of low frequency aberrations in driving resistant and refractory disease [20]. Promising candidate genes and rearrangement events continue to be identified, however their roles in neuroblastoma remain to be fully elucidated (Table 2). Low frequency mutations have been identified in *ARID1A/B*, *ATRX*, *LIN28B* and *TP53*, as well as genes in the MAPK and Rho-Rac signalling pathways, especially in relapsed neuroblastoma [20,21]. Outside of LIN28B amplification, most of these are inactivating mutations of tumour suppressor genes, and are therefore not directly targetable. LIN28B has been indirectly targeted through ornithine decarboxylase inhibition by Difluoromethylornithine, which has increased survival in high-risk neuroblastoma in combination with etoposide (NCT01059071). It is also currently being evaluated as a single agent in high-risk disease (NCT01586260, NCT02395666) [59].

The necessary preclinical and clinical investigation of many other aberrant genes is still lacking. *ARID1A* loss is known to promote neuroblastoma growth and resistance in vivo [60], and *ARID1B* alterations are also predictive of poorer patient outcome [61]. While there is little scope for targeted therapy in this context, the presence of an *ARID1A* mutation is a condition for PD-1 and dasatinib treatment in an ongoing phase II clinical trial for non-small cell lung carcinoma (NCT04284202). Loss of function mutations, or the deletion of *ATRX* has also been shown to sensitise neuroblastoma cell lines and PDXs to the current standard-of-care drug irinotecan, in combination with the PARP inhibitor olaparib [62]. However, even if there were targeted treatment options for each of these low-frequency mutations, the clinical benefits may only be relevant for a very small number of neuroblastoma patients.

**Table 2:** Frequency of notable genomic aberrations observed in neuroblastoma.

Gene	Function	Aberration	Frequency (%)			
			Diagnosis	Ref.	Relapse	Ref.
ALK	Receptor tyrosine kinase	Activating mutation	7–14.3	[20,39,63,64]	24.7	[63]
		Amplification	2–3.4	[63]		
ARID1A/B	Chromatin remodelling	Inactivating mutation	2–3	[65]	-	
ATRX	Chromatin remodelling	Inactivating mutation	1.8–5.5	[20,63,64]	11.1	
		Deletion	4–11	[20,64,66]	5.6	
FGFR1	Receptor tyrosine kinase	Mutation	0–1.7	[20,64]	9.3	
KRAS	Signalling protein	Mutation	0–1.7	[20,64]	1.9	
MYCN	Transcription factor	Amplification	16.5–37	[20,63,64,66]	16.3	
		Activating mutation	0.9–1.7	[20,64]		
NF1	Tumour suppressor	Inactivating mutation	0–2.2	[20,64]	5.6	
NRAS	Signalling protein	Activating mutation	0.8–2.6	[20,64]	7.4	
P53	Tumour suppressor	Inactivating mutation	0.8–3.5	[20]	7.4	
PTPN11	Tyrosine phosphatase	Activating mutation	1.3–2.9	[20,64]	0	
TERT	Telomerase reverse transcriptase	Inactivating mutation	13–25	[66,67]	-	

### 2.5. Targeting Epigenetic Aberrations

In line with the low rate of recurrent somatic mutations in neuroblastoma, it is now widely recognized that cancer progression generally requires both genetic and epigenetic involvement [68,69,70]. Accordingly, epigenetic dysregulation by aberrant DNA methylation and chromatin remodelling in neuroblastoma has also been correlated with patient prognosis [71]. Furthermore, the enigmatic clinical behaviour of neuroblastoma has also been hypothesized to be a product of fluid epigenetic states in neuroblastoma; further supporting the idea that neuroblastoma may be in part an epigenetically driven disease [72,73,74,75].

DNA methylation is a master epigenetic regulatory pathway governing genomic programming of a cell modulated by DNA methyltransferases (DNMTs) [76]. A loss of global DNA methylation leads to genome instability and this is commonly observed in neuroblastoma [77,78]. On the other hand, increased genomic methylation is associated with poor outcomes in neuroblastoma [79]. Interestingly, expression of DNMTs in neuroblastoma is equally paradoxical. While DNMT3B7 expression is associated with better clinical outcome [80], elevated DNMT3A and DNMT3B expression is observed in high-risk and cisplatin-resistant neuroblastoma tumours [81]. Taken together, this paradox represents both the opportunities and challenges associated with targeting the epigenome for therapeutic benefit. Promisingly, in vitro inhibition of DMNTs in neuroblastoma cells increases sensitivity to current standard-of-care chemotherapeutic agents such as cisplatin, doxorubicin and etoposide [82]. To date, two DNMT inhibitors, decitabine [83] and genistein [84], have progressed to phase I and phase II clinical trials, respectively. Lack of efficacy at tolerable doses has emerged as a recurrent theme in these trials, suggesting focus may need to shift towards targeting specific methylation events.

Histones are key DNA scaffolding proteins, which undergo post-translational modifications such as, acetylation, methylation, phosphorylation and sumoylation, that individually are able to dictate chromatin conformation and consequently, gene transcription [85]. The addition and removal of these histone marks is tightly regulated and an imbalance in their equilibrium is associated with cancer tumorigenesis and progression [85,86]. Histone methylation is a dynamic process controlled by histone methyltransferases (HMT) and histone demethylases (HDM) [87]. Mono-, di- and tri-methylation at Histone 3 Lysine 27 by the HMT EZH2 is known to be specifically increased in neuroblastoma [88], particularly in MYCN amplified tumours [89], and also associated with poor patient outcome [90]. As a result, EZH2 inhibitors have been investigated in a number of malignancies, including neuroblastoma [91]. DZNep is one EZH2 inhibitor which reduces EZH2 and induces apoptosis in colon and breast cancer cells, although its affinity for other HMTs has limited its application [92]. Another EZH2 inhibitor, EPZ6438 induced neuroblastoma cell differentiation through epigenetic modification of the TrkA promoter [90]. Preclinical testing of EPZ6438 in a cohort of paediatric solid tumours, including neuroblastoma, demonstrated anti-tumour activity in rhabdoid sarcomas only [93]. Completed phase I trials have determined single agent dosing of EPZ6438 in lymphomas [94], while other ongoing phase I trials have included advanced solid tumours (NCT01897571). A phase II study is currently investigating the use of EPZ6438 in EZH2 mutant tumours, including neuroblastoma (NCT03213665).

Histone acetylation is another key post-translational modification, which regulates vital cellular mechanisms including cell death, cell cycle progression and differentiation [95]. Histone acetyltransferases (HATs) add acetyl groups to lysine residues, inducing an “open” chromatin conformation and thus promoting transcription, while histone deactylases (HDACs) remove acetyl groups, condensing chromatin, resulting in transcriptional repression. A number of HDACs are implicated in tumour growth, cell survival and poor patient outcomes in neuroblastoma, while HDAC inhibitors have been extensively studied in numerous malignancies, including neuroblastoma [96]. As a result, HDAC inhibitors are currently under heavy interrogation in both preclinical and clinical trials. The broad spectrum HDAC inhibitor, vorinostat, is able to arrest cancer cell growth and induce apoptosis [97] and is effective in combination with current standard-of-care chemotherapy [98] and radiotherapy [99]. Promising results have been documented for numerous other HDAC inhibitors, however as of now, only vorinostat is being studied in phase II clinical trials [100]. Multiple reviews have recently been published addressing the potential for HDAC inhibition in neuroblastoma treatment [96,101,102], underlining HDACs as an attractive and promising target for therapeutic intervention.

## 3. Targeting Relapsed Neuroblastoma

Genetic analysis has also demonstrated that the sub-clonal heterogeneity of neuroblastoma tumours is reduced at relapse, suggesting that clonal selection drives the emergence of resistant subclones and is a significant factor in refractory or relapsed disease [11]. Notably, two landmark whole genome studies recently described the genomic landscape of recurrent and relapsed neuroblastoma and demonstrated these tumours had an increased spectrum of actionable mutations [10,11] (Table 2). Approximately 80% of relapsed neuroblastoma samples carried direct mutations or mutations in activators of the RAS-MAPK pathway [10]. A separate retrospective study found that 60% of relapsed tumours harboured mutations that are addressable with clinically validated targeted treatments [63]. Collectively, this evolving understanding of the spatiotemporal dynamic of neuroblastoma will have clinically relevant implications. New clinical studies such as NEPENTHE (NCT02780128) aim to exploit this by identifying actionable genetic mutations through next generation sequencing in relapsed or refractory neuroblastoma. Treatment arms will target ALK (Cretinib) as well as CDK4/6 (Ribociclib), MEK1 (Trametinib) and p53 (HMD201), which are not traditional therapeutic targets in neuroblastoma.

However, an emerging theme in clinical oncology is the need to focus efforts upon preventing relapse from occurring through improvements in first-line therapies, given the inherent therapeutic intransigence of relapsed, and often metastatic, tumours. This need is highlighted by Fletcher et al., who proposed that future clinical trials should be tailored to firstly recognise high-risk patients likely to fail standard-of-care therapy and then identify the actionable aberrations that may drive sub-clonal expansion, facilitating the treatment of these patients before relapse, where patient outcome is significantly poorer [13]. Clearly, there is an urgent need to develop biomarkers capable of providing patient-specific predictions of response to standard-of-care chemotherapy drugs and also optimizing the combinations of these drugs with the many emerging therapies described above. However, to develop this platform, significant advances in patient-specific modelling approaches will be needed, as well as detailed biochemical information about the mechanism of action for standard-of-care chemotherapy drugs to inform the development of these models.

## 4. Standard-of-Care Chemotherapy

As outlined above, high-risk patients that present with unresectable and/or metastatic disease are administered neoadjuvant induction therapy. Induction therapy entails high intensity cycles of combination of anthracyclines, alkylating agents, platinum agents, microtubule destabilisers and topoisomerase II inhibitors. While regimens vary around the world, the USA-based, Children’s Oncology Group outline six cycles of induction chemotherapy: cycles 1,2- topotecan and cyclophosphamide; cycles 3, 5- cisplatin and etoposide; cycles 4, 6- cyclophosphamide, vincristine and doxorubicin [103,104]. Protocols are similar in Europe where etoposide is also included in induction therapy [15]. If induction therapy is successful, it is followed by surgical resection of the tumour. This is typically accompanied by autologous stem cell transplantation to restore circulating blood cell count, GD2 neuroblastoma-specific immunotherapy, isotretinoin (12-cis-retinoic acid) which promotes neuroblastoma cell differentiation, and radiation therapy to destroy residual cancer cells [15] (Table 1).

The most significant challenge for high-risk neuroblastoma and indeed most cancers, remains the treatment of relapsed or refractory disease. Despite recent advances, to date there remains no curative treatment regimen and prognosis for these patients remains dire despite intense multimodal salvage therapy. The camptothecin analogues topotecan and irinotecan are commonly used in salvage therapy, with established activity against refractory [105,106,107] and relapsed neuroblastoma [108]. Irinotecan and temozolomide [108], and topotecan and cyclophosphamide combinations [109], have achieved 16% and 30% objective response, respectively, for relapsed or recurrent disease. Disease regression has also been observed with combinations of ifosfamide, carboplatin, and etoposide, with disease regression in 82% and 50% of relapsed and refractory disease, respectively [110]. Other treatment modalities are also being evaluated in this setting, including iodine 131-tagged MIBG radiotherapy and chimeric IL2-GD2 immunotherapy [111]. Thus, high-risk disease and refractory or recurrent disease present the greatest clinical challenge for neuroblastoma treatment despite the evolution of intense multi-agent and multi-modal therapy. For these patients, there is an urgent need for new treatment options to improve patient outlook.

Despite the wide-spread use of these chemotherapy agents, outside of their direct cellular targets, the exact mechanism of apoptosis induction remains poorly defined (Table 3). Although, to inform patient-specific predictions of drug response, the biochemical pathways utilized by each standard-of-care chemotherapy drug to induce apoptosis will need to be mapped in detail. For example, we have previously demonstrated that despite their disparate cellular targets, both the standard-of-care chemotherapy drugs doxorubicin and vincristine require activation of c-Jun N-Terminal Kinase (JNK) signalling to induce apoptosis in neuroblastoma cells [112]. As further outlined below, this study also demonstrated that ~40% of neuroblastoma tumours have an impaired ability to activate JNK signalling, suggesting that these two drugs would be predictably ineffective for these patients.

Therefore, the development of dynamic mathematical models that encapsulate the apoptotic pathways activated and required by these drugs will then allow the inclusion of individual tumour specific changes and the simulation of patient-level drug response. Further developments will also require more sophisticated approaches that can predict the optimal application of emerging and pre-existing therapies within context of multi-agent treatment regimens. However, many such approaches are already beginning to emerge, which are outlined below.

## 5. Personalized Models of Chemotherapy Response

Traditionally, a personalized medicine approach involves tailoring the use of specific targeted therapeutics to individual patients based upon the presence of an actionable genetic mutation within their tumour. However, the low frequency of actionable mutations within neuroblastoma means that there is little scope for a personalized medicine approach that will benefit the entire patient population. Recent advances with ALK and Trk inhibitors have demonstrated a potential to benefit subsets of high-risk neuroblastoma patients. In addition to this, clinical trials are also underway to match targeted therapies to the emergence of actionable mutations within relapsed neuroblastoma, but due to the established decrease in treatment efficacy in relapsed neuroblastoma, a focus on preventing relapse by optimizing induction therapy is desperately needed [13].

To achieve this, what is urgently needed is a paradigm in which standard-of-care chemotherapy drugs, and emerging broad-spectrum drugs, can be deployed as tailored induction therapy regimens based upon individualized predictions of drug response. To develop such a personalized medicine approach, it will firstly be necessary to understand the apoptotic mechanism of each individual drug and the mechanism of synergy between potential drug combinations. By incorporating this mechanistic data into predictive, mathematical models, patient-specific perturbations can then be introduced, allowing the simulation of drug response and predictions of optimal drugs and drug combinations.

To achieve this ambitious aim, advances in computational modelling approaches will undoubtedly be necessary. This requirement arises chiefly due to the highly complex nature of signalling networks, which often contain inter-linked pathways and regulatory structures that prevent an intuitive understanding of network states and functional outcomes [123,124,125,126]. Modelling approaches can certainly provide an avenue for both encapsulating and leveraging this complexity, and are beginning to emerge within the field of precision medicine [127,128]. There are several different approaches to modelling, ranging from descriptive statistical data-driven analyses to predictive mechanistic models. We have recently reviewed these approaches, offering a comprehensive outline of the strengths and limitations associated with each [129].

In the context of personalized medicine, mechanistic modelling approaches are most suitable due to their ability to facilitate the inclusion of the spatiotemporal aspects of intracellular signalling that are typically absent in other modelling approaches. Consequently, mechanistic models can provide more accurate in silico simulations of biological systems and the key regulatory structures that signalling networks utilize to ensure robust decision-making processes [130]. This characteristic is vital for the personalization of these predictive simulations through the inclusion of patient-specific data, which may represent differences in the expression level of network components or the impact of known genetic mutations. However, this requirement for extensive prior knowledge of network structures and dynamics greatly increases the experimental efforts required for their generation and calibration [131,132].

Nonetheless, the power of mechanistic models for a personalized medicine approach is already becoming apparent. For instance, we have previously demonstrated the prognostic significance and predictive capacity of a patient-specific modelling approach for neuroblastoma patients [112]. In this study we constructed an ordinary differential equation-based model of the drug-induced, apoptotic JNK signalling network in the context of neuroblastoma. By incorporating patient-specific variations in the expression levels of each component of the JNK signalling network we were then able to perform simulations of the ability of each individual tumour to activate JNK signalling, and by extension apoptosis, in response to chemotherapy. Importantly, this study demonstrated that impaired JNK activation in silico was a highly significant and independent indicator of poor overall survival for neuroblastoma patients. A clear consequence of this study’s findings was that the standard-of-care chemotherapy drugs for neuroblastoma, many of which are known to require JNK to activate apoptosis, would lack efficacy in patient tumours with impaired JNK signalling. Therefore, investigation into alternative chemotherapy drugs that do not require this specific apoptotic pathway should be a priority for improving outcomes for these patients.

A similar model was created to predict PD-L1 expression and hence susceptibility to checkpoint inhibitor immunotherapy in neuroblastoma [133]. An integrated signalling network was built from established databases covering PI3K-AKT, MAPK, mTOR and Ras signalling pathways. From this model, ALK activation was identified as a critical signalling event driving PD-L1 expression. ALK inhibition with crizotinib reduced PD-L1 expression in silico and was validated in vitro, and this was not observed with mutant ALK tumours, suggesting neuroblastoma tumours with *ALK* mutations may be good candidates for PD-1 checkpoint inhibitors such as nivolumab [133].

Previous studies from other cancer types have also demonstrated the potential for the development of models capturing variations in the expression level of key network components for personalized predictions of drug response and thus the tailoring of treatment strategies to individual patients. This includes previous models of the acquisition of resistance to endocrine therapy in breast cancer, based upon the expression of EGFR and HER2 [134] and the simulation of response to either continuous or intermittent treatment regimens [135]. This group recently published another computational cell cycle model of ER signalling and the cell cycle, rationalizing the response of breast cancer cells to combination treatment with endocrine therapy and cell cycle inhibition [136]. Specifically, by incorporating data on c-Myc and hyper-phosphorylated RB1 levels, this model was able predict the optimal combination treatment of oestrogen deprivation and CDK4/6 inhibition with palbociclib. The authors also demonstrated utility of this model in explaining acquired resistance to continuous sequential therapy [136].

Another example comes from a model of the interconnected network of BCL-2 family, BH3-only, and other apoptotic regulatory proteins in colorectal cancer, which was used to simulate the influence of tumour-specific changes in expression of these proteins on apoptotic signalling in response to chemotherapy and investigate the association of these predictions with clinical response [137]. An additional study in colorectal cancer focused alternatively on the EGFR and IGF1R pathways, and developed a mechanistic model that incorporated a perturbation dataset covering common alterations observed in this cancer type [130]. This approach facilitated cell-line specific predictions of network rewiring in response to EGFR inhibition, allowing the identification of tailored therapeutic interventions to prevent the acquisition of resistance to EGFR inhibitors. Within the same disease context, a cell line-specific logic model has also been constructed, encompassing 14 phosphoproteins under 43 treatment conditions, and used to predict sensitivity to a panel of 27 drugs [138]. This modelling approach was capable of predicting sensitivity of the cell lines for 14 drugs, 9 of which had no genomic biomarker of therapeutic response. This result quite elegantly demonstrates the clinical insight these models can provide, above and beyond identifying static genotypes which currently underpin personalized precision medicine approaches. More recently, this group coupled microfluidic-based ex vivo high-throughput screening of cancer biopsies with mathematical modelling to generate patient-specific models of intrinsic and extrinsic apoptosis [139]. This powerful combination of high-throughput tumour analysis and mechanistic modelling was able to identify personalized drug combinations for pancreatic cancer patients, based upon the varying apoptotic capabilities of each tumour. This approach certainly paves the way for adoption into other tumour types, including neuroblastoma, where a broadly applicable, but highly personalized approach to personalized medicine will be required to improve outcomes for all patients.

## 6. Conclusions

Clearly, an extensive amount of future work will be required to fully map and model the extended apoptotic pathways required for response to chemotherapy in neuroblastoma cells, along with a focus on the bespoke collection of patient data to facilitate the generation of patient-specific simulations of drug response. In addition to this, determining the functional significance of relevant somatic mutations within these pathways and incorporating these alterations within patient-specific simulations will be a bottleneck that may only be overcome with extensive experimentation. While daunting, the effort required to establish this modelling platform will yield valuable data on strategies to optimize induction therapy regimens for high-risk neuroblastoma patients, thereby improving response rates and reducing the incidence of relapse. However, these approaches may also provide insight into the mechanisms of chemoresistance that arise within relapsed/refractory neuroblastoma tumours, providing further avenues of research aimed at improving outcomes for this young patient population.

## Figures and Tables

**Table 3:** Current standard-of-care drugs and prospective drugs evaluated for the treatment of neuroblastoma and their respective targets and mechanism of action.

Current Drug	Drug Class	Target/s (*Gene*)	Ref. [113]
Carboplatin (Paraplatin)	Platinum Alkalating agent	DNA	[114]
Cisplatin (Platinol)	Platinum Alkylating agent	DNA DNA-3-methyladenine glycosylase (*MPG)* Alpha-2-macroglobulin (*A2M*) Serotransferrin (*TF*) Copper transport protein ATOX1 (*ATOX1*)	[114,115]
Cyclophosphamide (Neosar)	Nitrogen mustard Alkylating agent	DNA Nuclear receptor subfamily 1 group 1 member 2 (*NR1I2*)	[116]
Doxorubicin (Adriamycin)	Anthracycline DNA intercalator Topoisomerase II inhibitor	DNA DNA topoisomerase 2-alpha (*TOP2A*) Nucleolar and coiled-body phosphoprotein 1 (*NOLC1*)	[117,118]
Etoposide (VePesid)	Camptothecin Topoisomerase II inhibitor	DNA topoisomerase 2-alpha (*TOP2A*) DNA topoisomerase 2-beta (*TOP2B*)	[117,119]
Irinotecan	Camptothecin Topoisomerase I inhibitor	DNA topoisomerase 1 (*TOP1*)	[120]
Topetocan (Hycamtin)	Camptothecin Topoisomerase I inhibitor DNA intercalator	DNA topoisomerase 1 (*TOP1*) DNA	[121]
Vincristine (Vincasar)	Vinca alkaloid Anti-microtubule agent	Tubulin alpha-4a chain (*TUBA4A*) Tubulin beta chain (*TUBB*)	[122]

## Data Availability

Not Applicable.
